# Utilizing Google Trends to Track Online Interest in Elective Hand Surgery During the COVID-19 Pandemic

**DOI:** 10.7759/cureus.17313

**Published:** 2021-08-19

**Authors:** Kurt M Mohty, Nassim Lashkari, Daniel J Gittings, Jennifer A Bell, Milan Stevanovic, Luke T Nicholson

**Affiliations:** 1 Department of Orthopaedic Surgery, University of Southern California, Los Angeles, USA

**Keywords:** hand surgery, elective surgery, covid-19, coronavirus, google trends, google, orthopaedic surgery, carpal tunnel, trigger finger, ganglion cyst

## Abstract

Introduction

Elective hand surgery encompasses a large volume of orthopaedic cases annually. Carpal tunnel syndrome, ganglion cysts, and trigger digits are some of the most common pathologies treated by hand surgeons. In the midst of the COVID-19 pandemic, patient's interest in elective hand surgery for these conditions is uncertain. The objective of this study is to use Google Trends to track online interest in elective hand surgery in the United States during the COVID-19 pandemic.

Methods

Online search trends regarding elective orthopaedic hand surgery were obtained via Google Trends from November 2019 to November 2020. Three common hand pathologies in lay terms ("carpal tunnel," "‘ganglion cyst’ + ‘wrist cyst,’" and "trigger finger") and three hand surgery-specific keywords ("hand surgery," "carpal tunnel surgery," and "trigger finger surgery") were used as search terms. The search volume index (SVI) graphs for the United States for both sets of search terms were then generated from the Google Trends data and compared to the seven-day average of new COVID-19 cases per day as reported by the CDC. A separate SVI graph was then created for the search term "coronavirus” and was compared against both sets of search terms as above.

Results

Search trends for all elective hand pathologies and surgery-specific keywords remained constant from November 2019 to the beginning of March 2020 and then decreased significantly within a one-month period following the peak in COVID-19 cases the week of March 15, 2020. Search trends for these keywords increased to baseline levels over the next few months. The search trend for "coronavirus" demonstrated a small search volume index peak of 13 during January 2020 followed by the maximum peak of 100 during the week of March 15, 2020, corresponding to the decrease in search trends of elective hand surgery at that time.

Conclusions

Online interest in elective hand surgery remained constant prior to the COVID-19 pandemic; however, there was a marked decrease in search trends of elective hand surgery with the rise in daily reported COVID-19 cases, suggesting that patient's interest in elective hand surgery decreased with the onset of the pandemic.

## Introduction

The COVID-19 virus was declared a global pandemic disease by the World Health Organization (WHO) in March 2020, and as of July 2021, the WHO has reported over 4.1 million deaths related to COVID-19 globally [1,2]. The COVID-19 pandemic has had an unprecedented effect on health-care utilization of both emergent and elective procedures and posed an important challenge in the perioperative and postoperative management of the surgical patient. With the extraordinary burden that health-care systems nationally and globally are experiencing as a result of the pandemic, many elective surgical cases, including orthopaedic hand surgeries, have been delayed or withheld [3-6].

Elective hand surgery encompasses a large volume of orthopaedic cases per year. Several of the most common pathologies treated by hand surgeons include carpal tunnel syndrome, ganglion cysts of the wrist, and trigger digits [7-11]. Although a large portion of patients are treated successfully with nonoperative management, patients with persistent or severe symptoms may elect to pursue surgery [12,13]. In the midst of the COVID-19 pandemic, patient's interest in elective hand surgery for these common conditions is uncertain.

The utilization of the internet for the access to health-care information has become an increasingly important source of health-related information for consumers which has allowed individuals to obtain medical information rapidly, conveniently, and privately [14,15]. With the growth in the use of the internet as a primary source for the acquisition and dissemination of health information, internet search tools such as Google Trends have emerged as important sources of real-time information of online search patterns and can be useful in determining patient's interest in elective hand surgery over time [16-18]. The objective of this study is to use Google Trends to track online interest in elective hand surgery in the United States during the COVID-19 pandemic.

## Materials and methods

On November 23, 2020, online search trend data regarding elective orthopaedic hand surgery were obtained via Google Trends (https://trends.google.com/) from November 10, 2019 to November 8, 2020 [19]. Three common hand pathologies (carpal tunnel syndrome, ganglion cysts, and trigger digits) in lay terms and three hand surgery-specific keywords were used as search terms (Table 1). The search volume index (SVI) graphs for the United States for both sets of search terms were then generated from the Google Trends data and compared to the seven-day average of new COVID-19 cases per day as reported by the Centers for Disease Control and Prevention (CDC) (https://covid.cdc.gov/covid-data-tracker/#trends_dailytrendscases) [20]. A separate SVI graph was then created for the search term "coronavirus,” and this was then compared against both sets of search terms as above. Each term was queried separately and reported on a relative interest scale where 100 represents the maximum search volume and zero represents the minimum search volume. Data were obtained in one-week intervals.

**Table 1:** List of search terms queried using Google Trends

Google Trends search terms
"coronavirus"
"carpal tunnel"
"ganglion cyst" + "wrist cyst"
"trigger finger"
"carpal tunnel surgery"
"hand surgery"
"trigger finger surgery"

To determine the significance of differences in relative search interest after the surge of COVID-19 in the United States, the means and standard deviations of each term were calculated for the search volume indices two months before and after March 15, 2020. This time frame was selected to capture the drop in search trends after the COVID-19 surge without encompassing the return to baseline search volumes after this time point. An unpaired two-tailed Student’s *t*-test was used to compare differences in means before and after this date. P values of less than 0.05 were considered statistically significant. All statistical analysis was conducted in Excel 2016 (Microsoft, Redmond, WA, USA).

## Results

**Figure 1:**
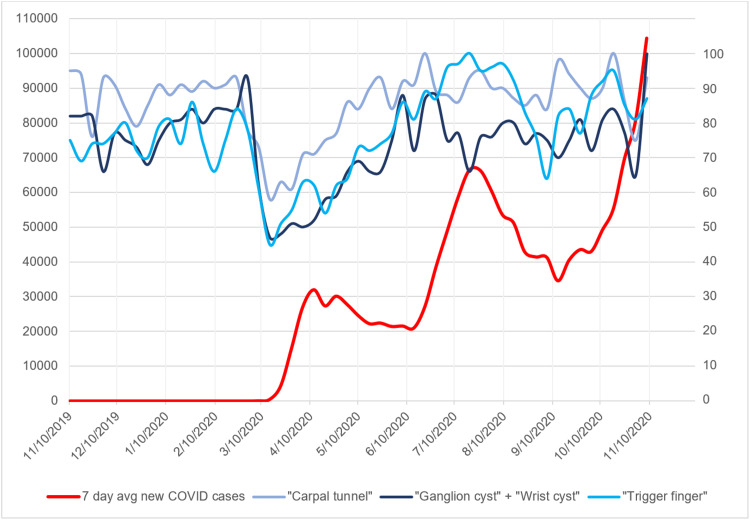
Seven-day average of new COVID-19 cases with search volume indices for hand pathology search terms Graph of seven-day average of new COVID-19 cases plotted against search volume indices for the hand pathology search terms “carpal tunnel,” “ganglion cyst” + “wrist cyst,” and “trigger finger.” x-axis represents time in months. Left-side y-axis represents number of new COVID-19 cases averaged over a seven-day period. Right-side y-axis represents relative search volume index for the above search terms.

**Figure 2:**
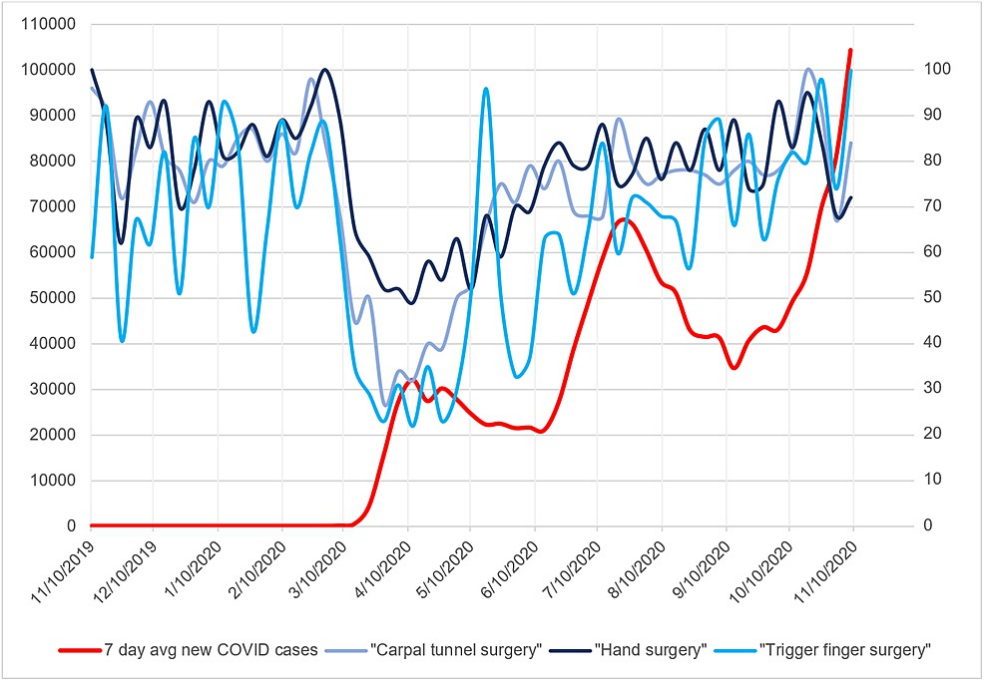
Seven-day average of new COVID-19 cases with search volume indices for hand surgery search terms Graph of seven-day average of new COVID-19 cases plotted against search volume indices for the hand surgery search terms “carpal tunnel surgery,” “hand surgery,” and “trigger finger surgery.” x-axis represents time in months. Left-side y-axis represents number of new COVID-19 cases averaged over a seven-day period. Right-side y-axis represents relative search volume index for the above search terms.

**Figure 3:**
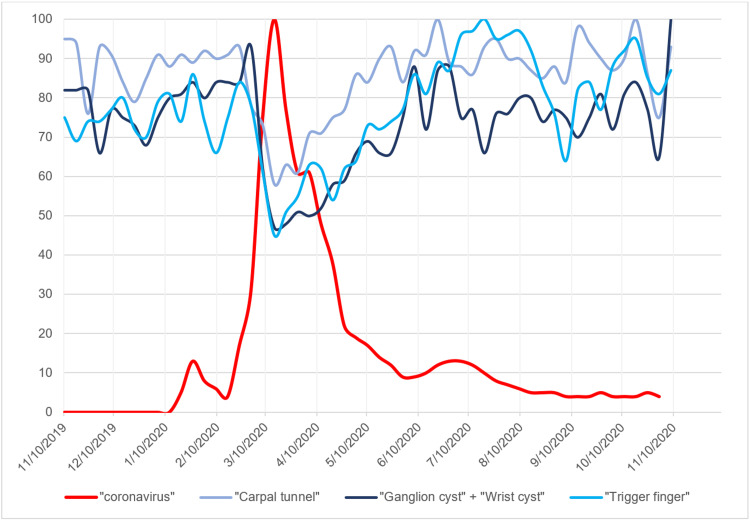
Search volume indices for the hand pathology search terms in addition to the search term “coronavirus” Graph of search volume indices for the hand pathology search terms “carpal tunnel,” “ganglion cyst” + “wrist cyst,” and “trigger finger” in addition to the search term “coronavirus.” x-axis represents time in months. y-axis represents relative search volume index.

**Figure 4:**
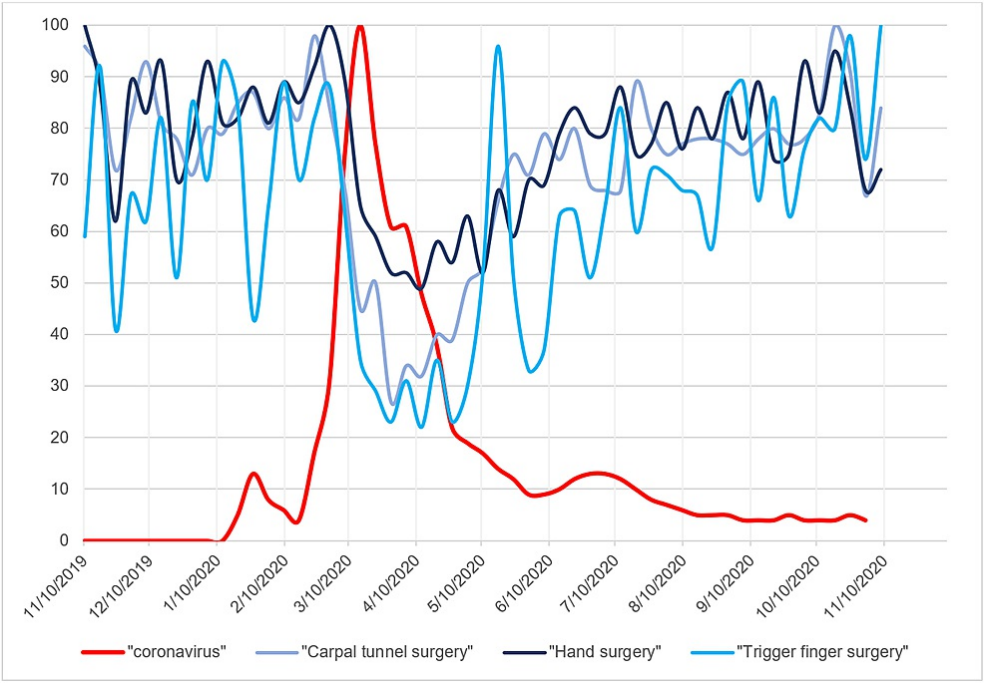
Search volume indices for the hand surgery search terms in addition to the search term “coronavirus” Graph of search volume indices for the hand surgery search terms “carpal tunnel surgery,” “hand surgery,” and “trigger finger surgery” in addition to the search term “coronavirus.” x-axis represents time in months. y-axis represents relative search volume index.

The search trends for all elective hand pathologies and hand surgery-specific keywords remained constant from November 2019 to the beginning of March 2020 (Figures 1, 2). However, with the sudden rise in reported COVID-19 cases beginning the week of March 15, 2020, all search trends decreased substantially. The search trend for "coronavirus" demonstrated a small SVI peak of 13 during January 2020 followed by the maximum peak of 100 during the week of March 15, 2020. This corresponded to the decrease in search trends of elective hand surgery at that time (Figures 3, 4). The baseline search interest averaged over a two-month period prior to the COVID-19 surge of March 15, 2020 was significantly higher than the search interest averaged over the two months following the surge (Table 2).

**Table 2:** Comparison of mean search volume indices two months before and after the COVID-19 surge of March 15, 2020 ^†^Reported as mean ± standard deviation. SVI: search volume index. P < 0.05 considered statistically significant.

Search terms	Average SVI two months prior to COVID-19 surge^†^	Average SVI two months after COVID-19 surge^†^	P value
"carpal tunnel"	87.1 ± 6.9	70.3 ± 8.7	0.001
"ganglion cyst" + "wrist cyst"	81.5 ± 8.2	53.9 ± 6.1	<0.001
"trigger finger"	74.8 ± 7.8	57.0 ± 6.4	<0.001
"carpal tunnel surgery"	83.8 ± 7.8	39.6 ± 7.9	<0.001
"hand surgery"	88.3 ± 5.7	56.5 ± 5.3	<0.001
"trigger finger surgery"	73.0 ± 14.8	28.5 ± 4.9	<0.001

The seven-day average of new COVID-19 cases rose to an initial peak of 32,034 at the beginning of April 2020 and slowly decreased to 20,994 at the beginning of June 2020 (Figures 1, 2). With this decrease in the seven-day average of new COVID-19 cases, the search trends for elective hand surgery and hand surgery pathology keywords slowly increased to baseline levels (Figures 1-4). Coinciding with the slow decrease in COVID-19 cases from April to June 2020, the relative search interest for the search term “coronavirus” rapidly decreased to an SVI of less than 10 by June 2020 and remained low for the remainder of the study period (Figures 3, 4). The search volume indices for hand pathology and elective hand surgery search terms remained at baseline levels even with the more extensive second peak of daily reported COVID-19 cases in July 2020 and the continual rise of cases in November 2020 (Figures 1, 2).

## Discussion

As the number of COVID-19 infections continues to grow worldwide, data on the impact of delayed medical and surgical care on patient-related morbidity and mortality are emerging [3,4]. In this study, we found that online interest in elective hand surgery remained constant from the fall of 2019 to early March 2020; however, there was a marked decrease in search trends of elective hand surgery with the sudden rise in daily reported COVID-19 cases in mid-March 2020, suggesting that interest in elective hand surgery decreased with the onset of the COVID-19 pandemic. Similar trends have been reported such as with public interest in total knee arthroplasty assessed through internet search queries decreasing with the onset of the pandemic to a rate less than half of that seen prior to the onset of the pandemic [16]. Although search interest initially decreased immediately after the sudden rise in reported COVID-19 cases, search trends for elective hand surgery and surgery pathology keywords returned to baseline values by June 2020 even with rising COVID-19 cases from July and fall of 2020. This suggests continued interest in elective hand surgery similar to levels seen prior to the pandemic. The exact reason as to why the search trends returned to baseline even with the subsequent COVID-19 waves cannot be fully elucidated given the nature of the retrospective study design. It is likely, however, that with the initiation of COVID-19 protocols in hospitals and surgery centres, elective cases could once again proceed in a safe fashion [3-6]. This might explain why search interest in elective hand surgery returned to baseline even with the rising COVID-19 cases in July and fall of 2020.

Carpal tunnel syndrome, ganglion cysts, and trigger digits are common hand pathologies that can have a considerable impact on a patient's quality of life. Carpal tunnel syndrome is the most common peripheral nerve entrapment syndrome worldwide with a prevalence in women almost four times higher than that in men and an estimated community prevalence of 1-5% [7,8,13,21,22]. Ganglion cysts are the most common soft-tissue tumours of the hand and wrist occurring most commonly in young adults with a slight female predominance [9,23]. Similar to carpal tunnel syndrome and ganglion cysts, trigger finger is most common among women in the fifth or sixth decade of life with higher reported prevalence in those with diabetes mellitus, rheumatoid arthritis, or amyloidosis [10,12,24,25]. Wildin et al. reported a 36% increase in primary care referral for elective hand surgery in the United Kingdom over the course of one decade with a referral rate of 112 per 100,000 patients per year for carpal tunnel syndrome and 55 per 100,000 patients per year for ganglion cysts [11]. With the continued increase in the community burden of these hand pathologies, patient's interest in elective hand surgery may continue to grow.

Analysis of Google Trends has previously been used in orthopaedics to measure public interest in a variety of procedures over time [16-18]. To our knowledge, this is the first study to describe the online search interest in elective hand surgery during the COVID-19 pandemic. Our findings are similar to those reported in other studies describing online search interest in elective total knee arthroplasty and stem cell injections for osteoarthritis of the hip and knee [16,18]. Similar to those studies, this study observed a significant decrease in online searches for elective orthopaedic surgery at the onset of the pandemic in the United States in March 2020. Our study, however, observed continued online search patterns throughout the summer and fall of 2020 with rates returning to those observed prior to the onset of the pandemic.

Although our data can aid in contributing to an understanding of current trends in interest in elective hand surgery, our study is not without several limitations. First, online internet searches may not accurately reflect public interest in elective hand surgery, and the association between interest in surgery and completion of surgery cannot be elucidated. Second, this study is also limited to Google Trends and may not accurately capture search trends using other internet search engines.

## Conclusions

In conclusion, Google Trends can serve as an important source of real-time interest in elective hand surgery. Online search trends of elective hand surgery significantly decreased with the sudden rise in daily reported COVID-19 cases, suggesting that interest in elective hand surgery decreased during this time. However, search interest in elective hand surgery returned to baseline levels during the summer and fall of 2020 which suggests that even though there was an initial decrease in patient's interest in elective hand surgery during the start of the COVID-19 pandemic, patients were still interested in addressing their pathology amidst the restrictions that many medical centres have enacted to slow the spread of the virus. Further studies are needed to determine if the COVID-19 pandemic and corresponding public interest in elective hand surgery correlate with delay in definitive surgical management, and future policy should focus on maintaining continuity of care and preparedness during times of increased strain on the health-care system.
